# Self-Efficacy After Life Skills Training: A Case-Control Study

**DOI:** 10.5812/nms.11691

**Published:** 2013-12-10

**Authors:** Fatemeh Rezayat, Nahid Dehghan Nayeri

**Affiliations:** 1 Department of Psychiatric Nursing, Tehran University of Medical Sciences, Tehran, IR Iran; 2 Department of Management, Nursing and Midwifery Care Research Center, Tehran University of Medical Sciences, Tehran, IR Iran

**Keywords:** Self-Efficacy, Students, Nursing, Life

## Abstract

**Background:**

Nursing students’ self-efficacy is a predictor for their educational progress. Students, who believe that they can be successful in their studies, are more confident. Therefore, many universities have focused on life skills training programs to improve the mental health of their students.

**Objectives:**

This study was conducted to evaluate and compare self-efficacy in two groups of nursing students of Tehran University of Medical Sciences (TUMS). One group of students was trained on life skill programs, and the second group was not trained on the issue.

**Materials and Methods:**

A case-control study was conducted on two groups of nursing students in TUMS in the late 2012. The case group (n = 112) had passed life skills training course, and the control group (n = 139) was not trained on the issue. Data was collected using a questionnaire containing 12 questions about demographic features, and the Sherer’s general self-efficacy questionnaire. Data analysis was performed using independent sample t-test, Chi-square, odds ratio, and Fisher’s exact test.

**Results:**

In the untrained and trained groups, 23% and 8% of the students had very high self-efficacy, respectively. The overall mean scores of self-efficacy were 41.99 ± 9.31 and 38.99 ± 10.48 in the trained and untrained groups, respectively (P = 0.015), and the higher mean score indicates lower level of self-efficacy. A significant difference was also found between the self-efficacy and family income (P = 0.029).

**Conclusions:**

The present study showed that life skills training program did not affect self-efficacy of nursing students. Perhaps, the methods used in education were influencing and then, more effective techniques such as role-play and group discussion should be substituted in life skills training.

## 1. Background

Self-efficacy reflects the judgments, beliefs, or expectations about a person’s capability to behave, engage, or implement actions in a given situation (1). People’s ability to fulfill a function depends on their beliefs rather than their skills (2). Nursing literature suggests that the growth of students’ self-efficacy would help to reduce the theory-practice gap (3). The sense of self-efficacy affects individuals’ performance both directly and indirectly through its effects on other determinants (4). Students’ self-efficacy may affect their ability to realize the educational objectives (5). The sense of self-efficacy can be changed by managing and manipulating the sources of perceptions (1). Students, who believe that they can be successful in their studies, indicate higher levels of excitement, attempt, and persistence in doing their homework, and are more confident. According to some studies, self-efficacy affects knowledge attainment, growth, and improvement of the skills (6, 7). People with high self-efficacy are more excited to learn and apply more strategies that are effective when dealing with problems (5). They can also manage their learning in a better manner, to complete their educational courses, and be prepared for their selected jobs (8). Self-efficacy is also related to performance and health status. In addition, high self-efficacy is associated with higher levels of competency and self-confidence that can cause a stronger feeling of well-being and control the stressing process in nursing students (9). People, who believe that potential dangers are not manageable, are considering some of the environmental aspects as dangerous elements. Because of such attitudes generated from lack of self-efficacy, they are anxious and disturbed. These people expect failure in their jobs, and withdraw against the challenges or even completely give up (10). In addition, self-efficacy plays the key role in development of critical thinking. Self-efficacy beliefs are as the main components of the behavior, and can predict critical thinking potentials (11). Some studies evaluated the effect of life skills on self-efficacy. However, because self-efficacy is culture-based (12), we need to evaluate it in different cultures. It has been reported that nursing students graduated with higher self-efficacy have the required independence and self-confidence to apply theory into practice (13). Self-efficacy makes relatively constant change in trainee’s behavior (2). Nursing students are more likely to face depression and distress due to the high workload, stresses related to the university, clinical practices, competition atmosphere, and their short free time to have fun. Self-efficacy can be learned; also it changes over the time. Regarding learning ability of self-efficacy, many universities have a particular focus on teaching life skills, and those skills required for improving the students health and self-efficacy (14). Life skills include capabilities which prepare people for a better resistance to the life challenges. These skills enable individuals to behave more properly and be more flexible in the society, and promote their self-esteem (15). Moreover, life skills training programs have a key role in promotion of mental well-being state, merit (16), assertiveness, self-confidence, and responsibility of the individuals (17). Tavakolizadeh and Ebrahimi (18) studied the effect of teaching of self-regulation learning strategies on self-efficacy, and found a significant increase in self-efficacy of the second grade of junior high school students. Moreover, the results of a study conducted by Ammentorp et al. showed that communication skills training resulted in an improvement in nurses and physicians’ self-efficacy (19). Despite studies conducted on the effect of life skills training on self-efficacy and other personal features (15-17, 20-22), no research has been conducted to evaluate life skills training program implemented in Tehran University of Medical Sciences, particularly about its effect on self-efficacy of nursing students. 

## 2. Objectives

This research was conducted to evaluate and compare self-efficacy in two groups of nursing students of Tehran University of Medical Sciences in 2012. One group of students has been educated on life skill programs and the second group were not trained. 

## 3. Materials and Methods

### 3.1. The Context of the Study

Tehran University of Medical Sciences has assigned life skills courses as a prerequisite for students since 2010. The program included sixteen two-hour sessions held once in a week by the university. Classes were held at different times and days of the week in the faculty. The educational content of these sessions included skills such as assertiveness, stress management, resentment control, effective communication, decision-making, problem solving, and negative temper control (dealing with depression). Some of these classes were managed by lecturing, group discussion, and role playing; while the other were held only using lecturing technique and a Master of Science in psychology. 

### 3.2. Design of the Study

A case-control study was conducted on two groups of nursing students of Tehran University of Medical Sciences in the late 2012. The first group (cases) had passed life skills training course, and the second group had no life skills training. The required sample size was considered as 228 students regarding the findings of Ammentorp et al. (19), and assuming that life skills training program would change self-efficacy at least 25% with confidence level of 95%. Total number of the participants at the beginning of the study were 270. However, as the total number of cases who entered the study were 112 participants. Then 139 non-trained students were entered as control group. Two hundred and fifty one of them filled the questionnaire and returned it back. The inclusion criteria for the case group were passing the courses for life skill training, and lack of any psychological illness, and the exclusion criterion was failure to return the questionnaire. The inclusion criteria for the controls were also the same except they did not pass the life skills course. The students who were not trained were in the first and sixth semesters, and those who passed were in their second and third semesters. Life skill programs only started two years ago, thus students of the first and sixth semester were untrained. The instrument used in this study was a self-report questionnaire containing 12 questions about demographic specifications and the Sherer’s et al. general self-efficacy scales. The Sherer et al. scale includes seventy five choice questions with five scores ranging from 1 (I completely disagree) to 5 (I completely agree). All questions scored from 1 to 5 except questions 3, 8, 9, 13, and 15 which were scored inversely. Based on choices for each question, the scores below 30 and above 72 indicated very high and very low self-efficacy, respectively. Experts’ panels in several studies have evaluated the content validity of the Sherer’s et al. questionnaire (6). Using α-Cronbach statistic, Ahadi et al. also computed the reliability coefficient of self-efficacy questionnaire as 0.81 (23). According to Barati, reliability of this scale was also 0.79 by calculating correlation between self-efficacy scale and internal-external control scale (23). Also, in the present study, reliability coefficient of the tool was determined as 0.867 using α-Cronbach test. 

### 3.3. Data Analysis

For data analysis, SPSS software package version 16 was used. Descriptive statistics were calculated for all variables. Also, independent sample t-test was used to compare self-efficacy mean scores in trained and untrained groups. Moreover, Chi-square and Fisher’s exact tests were used to determine uniformity of the both groups regarding demographic parameters, and determining the association between demographic parameters and self-efficacy.

### 3.4. Ethical Considerations

This study was approved by the Research Ethics Committee of Tehran University of Medical Sciences. Also permissions were obtained from the authorities in nursing school. Students were informed from the study objectives, and were free to participate in the study. The questionnaires were also anonymous, and all of the personal information was kept confidential. 

## 4. Results

From a total of 251 participants, 139 students were in the control group and 112 in the case group. No significant differences were observed between the case and control groups regarding demographics except for gender (Table 1). 

**Table 1.** Frequency Distribution Nursing Students Demographic Characteristics

Demographics^a^	Non-Educated, No. (%)	Educated, No. (%)	Test Results, Chi-Square	Test Results, P value
**Gender**			5.02	0.025
Female	83 (59.7)	82 (73.2)		
Male	56 (40.3)	30 (26.8)		
**Employment**			1.537	0.215
Yes	28 (20.3)	16 (14.3)		
No	110 (79.7)	96 (85.7)		
**Number of family members**			2.152	0.341
1 - 3	16 (11.9)	20 (18.3)		
4 - 5	96 (71.1)	70 (64.2)		
≥ 6	23 (17)	19 (17.4)		
**Family income**			0.554	0.758
Acceptable	60 (43.8)	45 (40.2)		
Relatively acceptable	67 (48.9)	60 (53.6)		
Not acceptable	10 (7.3)	7 (6.3)		
**Interest to field**			2.65	0.272
High	56 (40.9)	44 (39.3)		
Average	65 (47.4)	61 (55.4)		
Low	16 (11.7)	7 (6.3)		

^a^ A few participants did not respond to some questions (1 in employment, 7 in number of family members, 2 in family income and in interest to field)

Also, no significant differences were observed between self-efficacy of the students in the first and sixth semesters (t = 0.836, P = 0.45). As shown in Table 2, among demographic variables, only family income was significantly correlated with self-efficacy (P = 0.029). However, due to the similarity in the family income status in the two groups (Chi-square = 0.554, df = 2, P = 0.758), this parameter did not affect differently in the two groups. In the untrained and trained groups 23% and 8% of the students had very high self-efficacy, respectively (Table 3). The overall mean scores of self-efficacy were 41.99 ± 9.31, and 38.99 ± 10.48 in the trained and untrained groups, respectively (P = 0.015, t = 2.451). The higher mean score indicates lower level of self-efficacy. We also presented odds ratio in Table 3. Regarding the heterogeneity of the two study groups in term of gender, the self-efficacy mean scores in the two groups were compared in the two genders. Table 4 shows that no significant difference was observed between male students in the trained and untrained groups. However a significant difference was observed between female students in the two groups (P = 0.007). 

**Table 2.** Relation Between Some Demographic Parameters With Self-Efficacy Level

Demographic	Self-efficacy			Test results
	High, No. (%)	Average, No. (%)	Low, No. (%)	
**Gender**				Fisher’s Exact Test, P = 0.45
Female	116 (70.3)	42 (25.5)	7 (4.2)	
Male	58 (67.4)	24 (27.9)	4 (4.7)	
**Employment**				Fisher’s Exact Test, P = 0.08
Yes	28 (63.6)	14 (31.8)	2 (4.6)	
No	145 (70.4)	52 (25.2)	9 (4.4)	
**Number of family member**				χ^2^= 4.71, P = 0.59
1 - 3	23 (63.9)	13 (36.1)	0 (0)	
4 - 5	114 (68.7)	43 (25.9)	9 (5.4)	
≥ 6	32 (76.2)	9 (21.4)	1 (2.4)	
**Family income**				Fisher’s Exact Test, P =0.03
Acceptable	83 (79.1)	20 (19)	2 (1.9)	
Relatively acceptable	82 (64.6)	38 (29.9)	7 (5.5)	
Not Acceptable	8 (47)	7 (41.2)	2 (11.8)	
**Interest to field**				Fisher’s Exact Test, P = 0.19
High	76 (76)	22 (22)	2 (2)	
Average	84 (66.7)	35 (27.8)	7 (5.5)	
Low	14 (60.9)	7 (30.4)	2 (8.7)	

**Table 3.** Frequency Distribution of Self-Efficacy Level in Nursing Students in Each Group

Self-Efficacy Level^a^	Groups	
	Non-Educated, No. (%)	Educated, No. (%)
**Very high (< 30)**	32 (23)	9 (8)
**High (31 - 44)**	69 (49.6)	64 (57.1)
**Average (45 - 58)**	34 (24.5)	32 (28.6)
**Low (59 - 72)**	3 (2.2)	7 (6.2)
**Very low (> 72)**	1 (0.7)	0 (0)
**Total**	139 (100)	112 (100)

^a^ Odds ratio was calculated after integration of the first two lines as high (very high + high), and the next three lines as low (average + low + very low). Odds ratio = 1.42, CI _odds_ = 0.829 - 2.43.

**Table 4.** Mean and Standard Deviation of Self-Efficacy Separated by Gender

Gender	Group, Mean (SD)		Test Result			
	Non-Educated	Educated	T	CI	df	P
**Male**	39.84 (11.63)	41.13 (9.37)	-0.52	-6.2, 3.61	84	0.601
**Female**	38.24 (9.65)	42.31 (9.32)	-2.75	-6.98, -1.14	163	0.007

## 5. Discussion

The findings of this study indicated that self-efficacy in the trained group was significantly lower than the untrained group. Because the students in the trained group were in the first or sixth semesters, we checked self-efficacy inside the subgroups and no significant differences were observed between self-efficacy of the students in the first and sixth semesters. Thus, these subgroups were the same regarding the self-efficacy, and nursing education did not affect it. In this study, the results showed that about 72% of untrained students and 65% of the trained ones had high or very high self-efficacy. Karabacak et al. reported that most of the nursing students in their study had a high level of self-efficacy (24). Lauder et al. have also found that self-efficacy scores of the preregistration student nurses and midwives were similar to other population means (25). Peterson-Graziose et al. in a study on associate degree nursing students found that a total of 82% of these students reported high self-esteem, and 77% indicated high self-efficacy (26). Chemers et al., in a longitudinal study on first year university students’ self-efficacy, found that academic self-efficacy and optimism were strongly related to the students’ performance and adjustment (27). However the results of the current study contradict the findings of Tavakolizadeh and Ebrahimi (18), Ammentorp et al. (19), Alizadeh et al. (28), Hommes and VanderMolen (29), and Doyle et al. (30). Also these studies are different from our research regarding research population, sampling methods and sample size, length of intervention, educational content, and research tools. Tavakolizadeh and Ebrahimi also trained a group of high school students on self-regulation learning strategies, and reported that the students’ self-efficacy was increased after the interventions (18). In another study, the adolescent girls’ self-efficacy has significantly increased after encouragement training based on Adler’s theory (28). Moreover, Ammentorp et al. reported that self-efficacy of nurses and physicians was improved after training in communication skills (19). Similar results were reported by Hommes and Van der Molen (29) and Doyle et al. (30) who evaluated the effects of different methods of communication skills training and handling difficult communication situations on self-efficacy of nurses and psychology students. Research has shown that self-efficacy could be affected by learning, experience, and feedback. However, a combination of internal and external factors such as individual’s knowledge, skills, physical conditions, self-esteem, levels of stress, interpersonal environment, complexity of the activity, and the time of performance (20). The disagreement between the results of the present study and previous studies can be ascribed to the different methods applied, and to the context of the program (31). On other hand, the insignificant effect of the courses of the program in this study may be attributed to the type of the study. Because the current study was an observational one and the researchers had no control over the content or the methods. While, other studies were experimental and there were some controls over the applied content and teaching methods. However, teaching the interpersonal skills requires a combination of creative techniques to be effective. The findings of current study showed a correlation between family income and self-efficacy, so that students with sufficient family income showed higher levels of self-efficacy. Koparan et al. also reported a significant association between self-efficacy and sufficient family income (32). Some researchers have also reported that people living in lower social classes are typically faced with problems such as inappropriate housing, inadequate nutrition, and lack of proper entertainments, that all adversely affect their mental health (33). However, the finding of the present study did not show any association between self-efficacy and household number, birth order, having parents, education, and job of the parents, housing status and gender, which were in agreement with the results of the studies conducted by Hadi on hospital nurses in Karaj, Iran (34), and Arabian et al. who investigated the relation between self efficacy beliefs, mental health, and academic achievement among students (35). In conclusion, the present study showed that life skills training program in university did not affect self-efficacy of nursing students. Regarding the differences in applied approaches for teaching the trained groups, it is suggested to implement more effective techniques such as role-play and group discussion. The participants in this study were the students from a single faculty, and this may limit the generalizability of the results. Therefore, future studies are suggested with larger sample size and from a variety of majors and different universities in the country. 
